# C-954-13. Outcomes Associated with Transfer Status for Burn Patients: A National Analysis

**DOI:** 10.1093/jbcr/irag033.145

**Published:** 2026-04-06

**Authors:** Aren E Kurth, Christopher Rittle, Jacob M Dougherty, Zhaohui Fan, Raymond Jean, Naveen F Sangji

**Affiliations:** University of Michigan Burn Center, Ann Arbor, MI; University of Michigan, Ann Arbor, MI; Wayne State University School of Medicine, Detroit, MI; University of Michigan, Ann Arbor, MI; University of Michigan, Ann Arbor, MI; University of Michigan, Ann Arbor, MI

## Abstract

**Introduction:**

Burn injuries result in approximately 32 000 inpatient hospitalizations at burn centers annually in the United States. The uneven distribution of burn centers across the US requires some patients to initially seek care at facilities not equipped to care for burn injuries, which necessitates transfer to specialized burn centers. This study evaluates outcomes associated with the transfer status of a burn patient.

**Methods:**

A retrospective cohort study was conducted utilizing the Healthcare Cost and Utilization Project National Inpatient Sample (HCUP-NIS) data from 2016 to 2020. Individuals of all ages with an International Classification of Diseases 10th revision (ICD-10) diagnosis of burn injury were included. Facilities with average annual burn diagnosis admissions ≥50 patients/year were included to limit the analysis to facilities that could be considered burn centers. A weighted sample was used to create the cohort. Descriptive analysis and logistic regression were used to determine association of transfer status with outcomes of interest, including mortality, morbidity, and hospital length of stay (LOS). Statistical significance was set at *p*<.05.

**Results:**

The cohort included 100 770 burn patients admitted to facilities with ≥50 burn admissions per year. Patients who were more likely to be transferred in than present direct to the facility included those aged 0–17 (OR 1.62; 95% CI, 1.55–1.69); those with 50–79% total body surface area (TBSA) burned (OR 2.00; 95% CI, 1.88–2.12); patients from urban areas (OR 3.00; 95% CI, 2.29–4.04); and patients with chemical burns (OR 1.43; 95% CI, 1.33-1.55) and frostbite (OR 1.35, 95% CI, 1.23-1.48) (Fig. 1). Patients transferred to facilities with specialized burn care experienced longer LOS (mean 9.0 vs. 8.5 days; *p*<.001) than patients directly admitted to such facilities.

**Conclusions:**

There are differences in patient factors, etiology of injury, and outcomes amongst patients who are transferred to a facility treating burns after injury compared to patients who present directly to such a facility. These findings raise questions about access to burn centers, resources required for transfer, and the impact of expanded access to burn centers versus a hub-and-spoke model for treatment of burns.

**Applicability of Research to Practice:**

These data have implications for determining the ideal distribution of burn centers across the country to ensure equitable patient access to these centers.

**Funding for the study:**

The PI is funded by the American College of Surgeons Faculty Research Fellowship for their research on access to specialized burn care.

